# Comparative Assessment of Vessel Sealing Devices in Laparoscopic Salpingectomy of Captive *Papio hamadryas*

**DOI:** 10.1155/vmi/1865766

**Published:** 2026-01-31

**Authors:** Marta Guadalupi, Pietro Laricchiuta, Roberta Belvito, Claudia Piemontese, Francesco Staffieri, Luca Lacitignola

**Affiliations:** ^1^ Department of Precision and Regenerative Medicine, University of Bari Aldo Moro, Valenzano, Bari, Italy, uniba.it; ^2^ Zoosafari, Fasano, Brindisi, Italy

**Keywords:** contraception in nonhuman primates, laparoscopic salpingectomy, minimally invasive surgery, *Papio hamadryas*, vessel sealing devices

## Abstract

This prospective randomized clinical study aimed to evaluate the feasibility, safety, and surgical performance of laparoscopic salpingectomy as a method for permanent contraception in captive *Papio hamadryas*, with particular focus on comparing two vessel‐sealing technologies: a radiofrequency bipolar device (LigaSure Dolphin Tip) and an ultrasonic scalpel (Harmonic). Thirty‐two healthy female baboons (25 adults and 7 subadults), weighing between 4 and 15 kg—including six pregnant and fourteen in estrus—were randomly assigned to either the LigaSure (LS; *n* = 16) or Harmonic (HS; *n* = 16) group. All animals underwent bilateral laparoscopic salpingectomy using a standardized three‐port technique. Surgical data included installation time (from skin incision to port placement), salpingectomy time (from final trocar placement to salpinx retrieval), and total surgical time (skin‐to‐skin). Intraoperative complications and postoperative recovery were monitored clinically and behaviorally. All procedures were successfully completed laparoscopically without the need for conversion or major complications. In the LS group, the mean (± *SD*) installation, salpingectomy, and total surgical times were 7.75 ± 3.51, 9.75 ± 4.16, and 28.9 ± 9.74 min, respectively, while in the HS group, the values were 7.56 ± 3.08, 11.3 ± 5.25, and 25.8 ± 6.62 min. Although the HS group showed slightly longer salpingectomy times, differences between groups were not statistically significant. Pregnant animals tended to require longer surgical times due to reduced intra‐abdominal working space. Based on these results, laparoscopic salpingectomy was consistently feasible, safe, and effective across a range of body sizes and reproductive statuses. Both vessel‐sealing devices performed reliably, and the procedure was well tolerated in all cases. These findings support the use of laparoscopic salpingectomy as a minimally invasive, efficient, and reliable option for permanent sterilization in captive nonhuman primate populations.

## 1. Introduction

Effective control of captive nonhuman primates (NHPs) reproduction is essential in these populations to ensure social stability and animal welfare [1, 2].

Traditional surgical sterilization techniques such as ovariectomy result in irreversible endocrine disruption and may affect behavior and social interactions [3–5]. In contrast, salpingectomy, involving the complete removal of the oviduct while preserving ovarian function, has been proposed as a more conservative surgical alternative. It prevents fertilization while maintaining normal endocrine function and cyclicity [6, 7].

In veterinary medicine, the use of minimally invasive techniques such as laparoscopy has proven beneficial for reducing tissue trauma and improving surgical outcomes [8–11].

In a previous study on *Papio hamadryas*, Lacitignola et al. demonstrated that laparoscopic salpingectomy was a safe, effective, and reproducible procedure for population control in captive baboons [12]. The authors reported low intraoperative morbidity, preserved ovarian function, and rapid return to normal social behaviors following surgery. These findings strongly support the implementation of this approach in zoos and breeding colonies.

Building upon those results, the present study aimed to evaluate and compare the performance of two advanced laparoscopic devices—a bipolar radiofrequency vessel sealing system (LigaSure [LS]) and an ultrasonic energy device (Harmonic Scalpel)—during salpingectomy procedures in *Papio hamadryas*. Special focus was given to variables such as operative time, technical feasibility, intraoperative safety, and adaptability in animals of varying size and reproductive status.

We hypothesized that laparoscopic salpingectomy could be performed safely and effectively in both adult and subadult baboons using either device, even in subjects in estrus or pregnant. However, we expected to observe differences in surgical efficiency and handling characteristics between the two technologies, especially in challenging anatomical scenarios. The study also aimed to validate this method as a consistent and minimally invasive solution for ovary‐sparing sterilization in captive *Papio hamadryas*.

## 2. Materials and Methods

### 2.1. Animal Welfare

The zoo management (Leo 3000 S.p.a., at Zoosafari di Fasano, Brindisi, Italy) granted approval for the study via informed consent. Surgical procedures were performed by an experienced veterinary surgeon supported by a veterinary team comprising zoo staff and members of the Department of Precision and Regenerative medicine and Jonic Area at the University of Bari, “Aldo Moro.” The study received ethical approval from the Clinical Studies Ethics Committee of the Department of Precision and Regenerative medicine and Jonic Area (DiMePRe‐J), University of Bari, “Aldo Moro” (Approval no. 05/2020).

### 2.2. Inclusion Criteria

Subjects included were adults and subadults (> 2 years old), excluding younger animals and those with reproductive system pathologies. Pregnancy was not an exclusion criterion in the context of this population‐management program; postponing procedures in gravid females would have required additional capture and chemical restraint and/or prolonged re‐handling, with potential welfare implications in a social zoological setting, Overall, this management‐based approach is consistent with previously published evidence supporting the feasibility of minimally invasive tubal sterilization in pregnant NHPs and with major guidelines indicating that laparoscopy can be performed during pregnancy when appropriately adapted (e.g., tailored entry and low‐pressure pneumoperitoneum) [13, 14].

### 2.3. Capture and Anesthesia

Three days prior to surgery, with assistance from the zoo keepers, a harem was isolated from the main group in a separate enclosure. Each animal was individually identified by a unique microchip number. Animals were fasted for 15 h and water withheld for 8 h before surgery. Animals were immobilized in a trap cage and manually injected with tiletamine‐zolazepam 2.5 mg/kg (Zoletil, Virbac S.r.l., Milan, Italy) and medetomidine 0.03 mg/kg (Domitor, Zoetis s.r.l., Rome, Italy). A 21‐gauge intravenous catheter was placed in the cephalic vein, and anesthesia was induced with intravenous propofol, and the airway was secured using an appropriately sized laryngeal mask; anesthesia was then maintained with isoflurane in oxygen. Anesthesia monitoring included heart rate, non‐invasive blood pressure, oxygen saturation (SpO_2_), capnography, and body temperature. Anesthesia was maintained with isoflurane. A single dose of meloxicam 0.2 mg/kg (Metacam, Boehringer Ingelheim, Milan, Italy) was administered to all subjects. Rescue analgesia with fentanyl 2 μg/kg IV (Fentadon, Dechra Veterinary Products, Turin, Italy) was provided in case of sudden changes in heart rate, respiratory rate, or blood pressure. Cefazolin 20 mg/kg IV was administered within 30 min prior to surgery. At the end of the procedure, isoflurane administration was interrupted and animals were assisted during recovery.

### 2.4. Surgical Procedure

**FIGURE 1:**
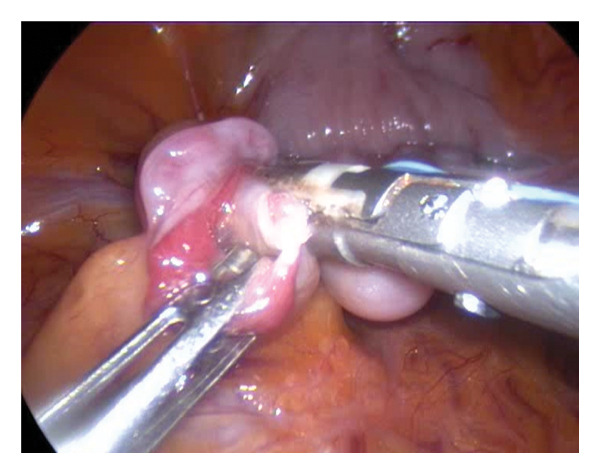
Right salpinx dissection during laparoscopic salpingectomy. The fimbrial end of the right oviduct is grasped and retracted in preparation for dissection. A bipolar vessel sealing device (LigaSure) is positioned to perform hemostatic transection of the mesosalpinx. Note the clear exposure of the anatomical structures and the atraumatic handling of the fimbria to preserve ovarian blood supply.

**FIGURE 2:**
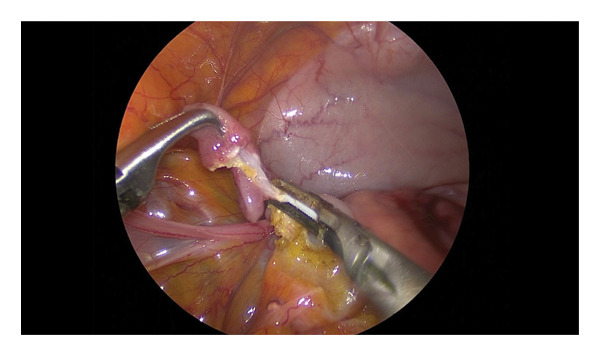
Right salpinx dissection using an ultrasonic scalpel (Harmonic). The fimbrial portion of the right oviduct is elevated and dissected with an ultrasonic energy device (Harmonic), allowing for simultaneous cutting and coagulation of the mesosalpinx. The image highlights the precise dissection plane and minimal lateral thermal effect, characteristic of ultrasonic technology.

Hair was clipped from the xiphoid process to the pubis. Skin asepsis was achieved with 4% chlorhexidine scrub alternating with alcohol (Dempol, Ecolab s.r.l., Milan, Italy). Animals were placed in dorsal recumbency on a tiltable surgical table and securely restrained with straps. A 5‐mm skin incision was made just caudal to the umbilical scar. Abdominal access was obtained using a modified Hasson technique [15] (Endopath XCEL Trocars, 5, 75 mm long J&J Ethicon Endo Surgery, Milan, Italy). An atraumatic Kelly clamp was used to elevate the abdominal wall. CO_2_ pneumoperitoneum was established through the access cannula at a flow rate of 1 L/min with a maximum pressure of 8 mmHg. Proper cannula placement and trocar positioning were confirmed under direct laparoscopic visualization using a 5‐mm 30° telescope (Karl Storz Endoskope, Tuttlingen, Germany). The abdominal cavity was inspected for inadvertent organ perforation or bleeding. Two lateral paramedian ports (Endopath XCEL, 75 mm long) were placed 3‐4 cm laterally at the same level to the median port under laparoscopic guidance, taking care to avoid injury to the superficial epigastric vessels. A 5‐mm Babcock forceps was introduced through the left port and a bipolar radiofrequency vessel sealing device (LS, 5 mm, Dolphin tip, Medtronic, Milan, Italy) or an ultrasonic laparoscopic device (ETHICON Ultracision Harmonic Scalpel) through the right port. The table was tilted to a 30° Trendelenburg position. The descending colon, small bowel loops, and omentum were gently retracted cranially to expose the pelvic organs. The urinary bladder was identified first, followed by the uterus. Ovaries and salpinges were identified and grasped. The salpinges were suspended with the Maryland forceps to expose vessels and mesosalpinges. The vessel sealing instrument was applied parallel to the salpinx, which was transected near the fimbriae attached to the ovary, approximately 3‐4 mm from the uterine insertion (Figures 1 and 2). The excised salpinx was retrieved through the port. The procedure was repeated on the contralateral side. CO_2_ insufflation was discontinued, laparoscope and gas evacuated, and trocars removed. Port sites were closed in two layers: the abdominal fascia was apposed with 3‐0 PDS using simple interrupted sutures, and the skin was closed with the same absorbable material to avoid postoperative suture removal and minimize recapture‐related stress.

### 2.5. Clinical and Surgical Variables

Age (adult or subadult) and weight (kg) were recorded with the mean ± standard deviation (SD). Estrous status was assessed by external genital swelling scored on a 0–4 scale as previously described [16]; scores 1–4 indicated estrus. Surgical variables recorded included total surgery time (from first skin incision to last suture), laparoscopic port installation time (from skin incision to last trocar placement), and salpingectomy time (from last trocar placement to retrieval of the second excised salpinges). Number of attempts to correctly place trocars, accidental organ injuries, and intraoperative complications were also documented.

### 2.6. Postoperative Care

Following extubation, animals were placed in a quiet, isolated enclosure to recover from anesthesia. Once fully recovered, baboons were reintegrated into the harem group. Postoperative monitoring included behavioral observations for social and individual changes, grooming, feeding, climbing ability, and signs of pain or stress. Antibiotic therapy was administered for surgeries exceeding one hour duration. Animals were released back into their habitual environment within four hours after recovery. Physical and clinical examinations were avoided postoperatively to prevent recapture‐related stress. One adult female was recaptured one week postsurgery for unrelated reasons and showed complete wound healing without infection.

### 2.7. Statistical Analysis

All statistical analyses were performed using Jamovi statistical software (The Jamovi Project, Version 2.3). Descriptive statistics were used to summarize the data. Continuous variables are reported as mean ± SD. Group comparisons for operative parameters were conducted using one‐way analysis of variance (ANOVA). A *p* value of less than 0.05 was considered statistically significant.

To evaluate the learning curve, salpingectomy time was plotted against the chronological order of surgeries. A piecewise linear regression model was applied to identify a breakpoint in the trend, representing a shift from the initial learning phase to a plateau of proficiency. In parallel, a cumulative sum (CUSUM) analysis was conducted to assess cumulative performance over time.

## 3. Results

### 3.1. Study Population

A total of 32 subjects were included: 25 adults and 7 subadults, with mean weight 10.38 kg (range: 4–15 kg and SD: 2.47 kg). Six females were pregnant; fourteen adults were in estrus. The remaining 12 adults and subadults were not in estrus. Animals were randomly assigned into two groups of 16 individuals each.

### 3.2. Surgery

All laparoscopic procedures were completed successfully without conversion to open surgery. No rescue analgesia was required.

The mean port installation time was 7.75 ± 3.51 min (range: 3–15) in the LS group and 7.56 ± 3.08 min (range: 4–17) in the Harmonic (HS) group. No intraoperative complications such as intestinal or bladder perforation were observed. Additionally, no moderate or severe hemorrhages occurred. Mean bilateral salpingectomy time was 9.75 ± 4.16 min (range: 4–21) in the LS group and 11.3 ± 5.25 min (range: 3–23) in the HS group. The overall mean total surgical time was 28.9 ± 9.74 min (range: 16–50) and 25.8 ± 6.62 min (range: 18–40) for the LS and HS groups, respectively (Table 1). Although no statistically significant difference was observed between devices in terms of salpingectomy time, a trend toward reduced operative duration was noted with increasing surgical experience. Moreover, based on the Tukey post hoc test, a statistically significant difference in salpingectomy time was detected between pregnant and estrus animals (mean difference = −7.00 min; *p* = 0.009) and between pregnant and nonestrus animals (mean difference = −8.78 min; *p* = 0.002), indicating longer operative times in gravid subjects.

**TABLE 1:** Descriptive statistics for body weight and procedural times by vessel‐sealing device.

Descriptives	Device	Weight	Installation time	Salpingetomy time	Total surgery time
Mean	LigaSure	9.63	7.75	9.75	28.9
	Harmonic	11.2	7.56	11.3	25.8

Standard deviation	LigaSure	2.87	3.51	4.16	9.74
	Harmonic	1.59	3.08	5.25	6.62

Minimum	LigaSure	4.00	3	4	16
	Harmonic	10.0	4	3	18

Maximum	LigaSure	14.0	15	21	50
	Harmonic	15.0	17	23	40

*Note:* Values are reported as mean, minimum, and maximum for subjects treated with LigaSure or Harmonic. Variables include body weight and operative time components (installation time, salpingectomy time, and total surgery time). Times are expressed in minutes; body weight is expressed in kilograms.

Abbreviation: standard deviation, SD.

### 3.3. Learning Curve Analysis

**FIGURE 3:**
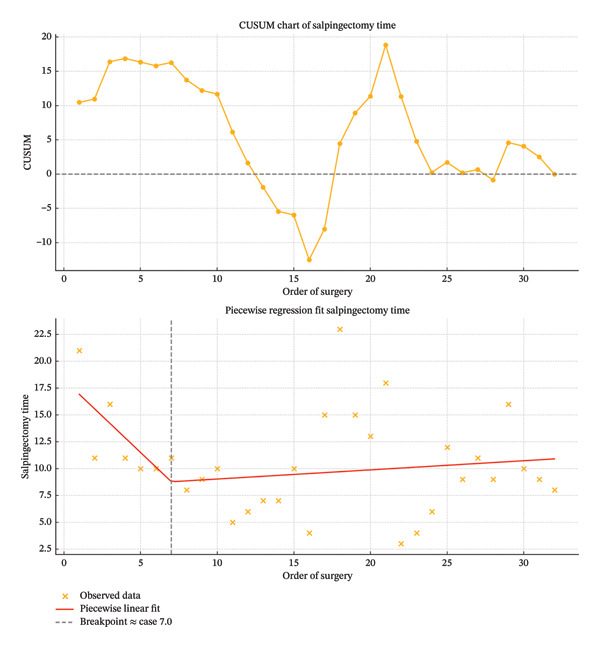
CUSUM analysis and piecewise regression of salpingectomy time across consecutive procedures. Top: cumulative sum (CUSUM) chart of salpingectomy time demonstrating performance trend across 32 consecutive laparoscopic salpingectomies. A learning curve is suggested by an initial upward deviation followed by a downward trend and stabilization around the zero line. Bottom: piecewise linear regression model showing a breakpoint at Case 7, indicating the end of the initial learning phase. A decreasing trend in surgical time is observed up to the breakpoint, followed by a flatter curve. Orange crosses represent observed salpingectomy times; the red line indicates the fitted regression; and the vertical dashed line marks the estimated breakpoint. Together, these plots support the presence of a learning effect with improved efficiency after the first seven cases.

A total of 32 laparoscopic salpingectomy procedures were evaluated to assess surgical performance and the learning curve. A clear decrease in salpingectomy time was observed during the initial phase of the study. The mean salpingectomy time progressively declined from higher values in the first procedures to a more stable range after the 7th case, as demonstrated by piecewise linear regression analysis. This model identified a significant breakpoint at approximately the 7th surgery, indicating a transition from the learning phase to a phase of improved proficiency. The CUSUM analysis further confirmed this trend, showing a steady cumulative reduction in deviation from the mean until around the 15th case, after which the curve plateaued and stabilized. A secondary increase in surgical time was noted between Cases 18 and 21 and again in Case 29. These cases corresponded to pregnant individuals, in whom anatomical changes and limited intra‐abdominal space likely contributed to prolonged salpingectomy times. Overall, the learning curve suggests that operative efficiency improves significantly after the initial 7 procedures and stabilizes thereafter, with pregnancy representing a relevant confounding factor in surgical duration (Figure 3).

### 3.4. Postoperative Evaluation

All animals recovered from anesthesia uneventfully and were reintegrated into the harem. Normal social and individual behaviors were observed, including grooming, feeding, and climbing within minutes after recovery, without signs of pain or stress. One week postsurgery, no reluctance to move, anorexia, isolation, or behavioral abnormalities were detected. Animals were returned to the main group. No postoperative physical examinations were performed to avoid additional stress from recapture. All pregnant females successfully completed gestation and carried their pregnancies to term.

## 4. Discussion

This study investigated the feasibility, safety, and performance of laparoscopic salpingectomy as a contraceptive strategy in captive *Papio hamadryas*, with particular focus on the comparative use of two vessel sealing technologies: the LS and the Ultracision Harmonic Scalpel. Key operative parameters, including port placement time, salpingectomy duration, and total surgical time, were evaluated. All procedures were successfully completed in adult and subadult females, including pregnant individuals, without intraoperative complications.

Instrument handling was effective even in small subjects weighing as little as 4 kg, consistent with findings in other NHPs using 5‐mm laparoscopic instruments. The use of short cannulas (75 mm) minimized inadvertent displacement, particularly in smaller individuals. Salpingectomy was performed with complete excision of the oviduct from fimbriae to the uterotubal junction. Both devices proved effective in achieving this, supporting their safety and reliability, as reported in other species [17, 18]. Although no statistically significant differences were observed between the two devices, the Harmonic Scalpel may allow for finer tissue control during mesosalpinx dissection, potentially due to its smaller jaws and reduced vibration. While this perceived advantage was not objectively measured, the surgeon reported greater maneuverability and precision with the HS, acknowledging the subjective nature of this impression.

Intraoperative bleeding was minimal and adequately managed by both instruments. Although thermal spread was not directly measured, prior studies have demonstrated reduced lateral thermal damage and better histological preservation with ultrasonic energy compared with bipolar radiofrequency devices [19, 20]. Importantly, no thermal injury to adjacent reproductive structures was observed, and ovaries and uterus remained uncompromised.

Operative times were comparable between groups. Mean total surgical time was 28.9 min for LS and 25.81 min for HS, with no statistically significant differences. Salpingectomy duration was slightly longer in the HS group (11.31 vs. 9.69 min), potentially reflecting the greater proportion of gravid females in this cohort, as pregnancy is known to reduce intra‐abdominal working space and increase technical difficulty.

Although advanced vessel sealing devices have been widely adopted across multiple surgical disciplines due to their technical advantages—such as reduced lateral thermal spread, integrated cutting mechanisms, and real‐time impedance feedback—their clinical superiority over conventional systems remains unproven in several contexts [21]. In human gynecologic surgery, current literature suggests that while these systems may reduce intraoperative blood loss and shorten operative time, these benefits do not consistently translate into clinically significant outcomes [22]. Meta‐analyses and controlled trials have failed to demonstrate a substantial reduction in complication rates, postoperative recovery times, or long‐term morbidity associated with their use [18]. Consequently, despite their growing popularity and sophisticated features, advanced vessel sealing devices selection often remains a matter of surgeon preference, resource availability, and economic considerations rather than robust outcome‐driven indications, even in veterinary surgery [17].

This study highlights a steep and well‐defined learning curve for laparoscopic salpingectomy. A significant improvement in operative efficiency was achieved within the first seven cases, as confirmed by CUSUM analysis and piecewise regression, which identified a breakpoint at case seven. This is consistent with previous findings in both human and veterinary laparoscopic surgery, where the most critical learning phase typically occurs during the initial procedures [23–25]. The low complication rate and operative efficiency observed are also likely influenced by the team’s prior experience with both laparoscopic techniques and energy‐based devices.

Notably, the HS group included five pregnant individuals (Cases 18–21 and 29), potentially introducing variability in operative times due to anatomical constraints imposed by the gravid uterus. Although pregnancy may reduce intra‐abdominal working space and increase technical difficulty, minimally invasive tubal sterilization has been reported as feasible in pregnant NHPs when performed with appropriate technique and monitoring. In human surgery, major guidance indicates that laparoscopy can be performed during pregnancy when clinically indicated, provided that abdominal entry is tailored to anatomy, physiologic monitoring is ensured, and pneumoperitoneum pressures are adjusted to maternal physiology. In the present study, a conservative maximum pressure of 8 mmHg was adopted [13, 14]. Nevertheless, because pregnancy was not an exclusion criterion and was unevenly distributed between groups, it may have acted as a confounding factor in comparisons of operative time and device‐related performance. Nonetheless, all pregnant subjects carried their pregnancies to term without obstetric or neonatal complications. This outcome strongly supports the safety of laparoscopic salpingectomy during pregnancy when performed with appropriate technique and insufflation settings [26]. To our knowledge, this is the first controlled report of successful laparoscopic salpingectomy in pregnant *Papio hamadryas*.

No postoperative complications were observed in relation to behavior, mobility, or feeding. However, systematic postoperative evaluations were intentionally omitted to avoid stress associated with chemical restraint, limiting follow‐up to group‐based behavioral assessment. Furthermore, the absence of long‐term follow‐up restricts conclusions regarding the permanence of sterilization, risk of adhesions, or late complications. Additionally, no validated postoperative pain scoring systems were used although animals resumed normal behaviors promptly and exhibited no overt signs of discomfort.

The study design also lacked stratified randomization based on estrous status or pregnancy. The unequal distribution of pregnant animals, predominantly in the HS group, represents a relevant confounding factor that may have influenced surgical times. Future studies should consider stratified allocation to ensure better homogeneity and allow a clearer comparison of device performance under standardized conditions.

In summary, this study supports and extends previous findings by Lacitignola et al. [12], confirming that laparoscopic salpingectomy is a safe, effective, and minimally invasive contraceptive option in captive *Papio hamadryas*. Moreover, it demonstrates that both vessel sealing systems are reliable for this procedure and that proficiency can be achieved after a limited number of cases. These results lay the foundation for further research and refinement of laparoscopic sterilization protocols in NHPs, particularly in the context of population management and welfare in zoological settings.

## Funding

The research did not receive specific funding. Open access publishing facilitated by Universita degli Studi di Bari Aldo Moro, as part of the Wiley ‐ CRUI‐CARE agreement.

## Conflicts of Interest

The authors declare no conflicts of interest.

## Data Availability

All relevant data generated or analyzed during this study are included within the manuscript. No additional datasets were generated or required for this work.
